# Dual functional genomics reveals a broad and convergent landscape of asciminib resistance in BCR::ABL1

**DOI:** 10.1186/s13073-026-01679-x

**Published:** 2026-06-27

**Authors:** Ivan Sokirniy, Zeyu Yang, Justin Pritchard

**Affiliations:** 1 Huck Institutes of the Life Sciences, The Pennsylvania State University, University Park, PA 16802 USA; 2 Department of Biomedical Engineering, The Pennsylvania State University, University Park, PA 16802 USA

**Keywords:** Asciminib, Drug resistance, Deep mutational scanning, Base editing, Epistasis, BCR-ABL, BCR:ABL1, CML, SH3, SH2

## Abstract

**Background:**

Drug resistance is a constantly evolving challenge. The allosteric inhibitor asciminib is a novel therapy for chronic myelogenous leukemia (CML) that targets the myristoyl pocket of the BCR::ABL1 kinase. While it can overcome resistance to active-site inhibitors like imatinib, new resistance mutations to asciminib are emerging. The complete landscape of these mutations, particularly those outside the kinase domain or those arising from epistatic interactions between mutations, are not well understood.

**Methods:**

This study employed a dual functional genomics approach in CML cell line models. A high-throughput adenosine base editing (ABE) screen was used to identify broad hotspots of asciminib resistance across the entire BCR::ABL1 protein. Deep mutational scanning (DMS) was then used to create a high-resolution map of all possible amino acid changes within these hotspots. An “edit-on-edit” screen was performed to investigate epistasis by introducing a library of mutations into a cell line that was pre-edited to incorporate the common imatinib-resistance mutation, Y253H. Finally, a novel Förster resonance energy transfer (FRET) biosensor was developed to measure the conformational state of BCR::ABL1 in live cells and link it to drug sensitivity.

**Results:**

The screens identified 279 asciminib resistance mutations and revealed resistance hotspots distributed across the SH3, SH2, and kinase domains, in contrast to imatinib resistance, which is largely confined to the kinase domain. The study uncovered a potent epistatic interaction between a mutation in the SH3 domain (V73A) and a mutation in the kinase domain P-loop (Y253H), which synergistically conferred high-level resistance. The FRET biosensor demonstrated that asciminib resistance mutations tend to destabilize the “closed” inactive conformation of the ABL1 kinase.

**Conclusions:**

The landscape of asciminib resistance is broader and more complex than previously appreciated, involving mutations across multiple domains that disrupt ABL1 autoinhibition. Epistasis between mutations acquired during sequential therapies can create unexpected and potent resistance. However, these diverse genetic resistance mechanisms converge on a single biophysical measurement of the openness of the active ABL1 conformation. This provides a unified framework for understanding asciminib resistance and underscores the need for routine clinical resistance monitoring to include the SH3 and SH2 domains in first line and later line therapy.

**Supplementary Information:**

The online version contains supplementary material available at https://doi.org/10.1186/s13073-026-01679-x.

## Background

Allosteric regulation, an ancient and ubiquitous mechanism controlling enzyme function, involves modulation of enzyme activity by effector molecules binding at sites distinct from the active site within a single protein. ABL1 possesses two regulatory domains: the SH3 and SH2 domains. Binding of the myristoylated N-terminus of ABL1 to a hydrophobic pocket within the kinase domain’s C-lobe triggers a conformational change. This interaction induces the SH3 and SH2 domains to clamp onto the kinase domain, effectively locking it in an inactive state [1–3].

The BCR::ABL1 fusion protein, resulting from a chromosomal translocation that truncates the ABL1 N-terminus, disrupts the allosteric regulation of ABL1 kinase, induces dimerization, and activation [2–4]. Imatinib, a competitive inhibitor, binds the active site of BCR::ABL1 [5, 6]. While effective for many patients, resistance can arise due to mutations primarily in the kinase domain [7–10]. Asciminib represents a novel approach to ABL1 inhibition. This first-in-class allosteric ABL1 inhibitor mimics the myristyl group of the physiological ABL1 N-terminus, inducing allosteric autoinhibition of the kinase by binding the ABL1 myristate binding site in C-lobe of the kinase domain [11]. Importantly, asciminib has demonstrated the ability to overcome key resistance mutations that render prior generations of active site inhibitors ineffective [12, 13]. However, new resistance mutations to asciminib, such as ABL M244V, I502L and V468F, have been identified within ABL1 kinase [14–17]. Moreover, in spite of early reports that argued that active site resistance is completely independent from myristate pocket resistance [18], recent clinical and preclinical evidence suggest that F359X mutations are resistant to inhibitors targeting the active site and the myristate pocket [19]. Whole domain deletions of the SH3 or SH2 domains are also known to confer potent asciminib resistance, suggesting the potential for resistance mechanisms outside of ABL1 kinase domain [20, 21]. Despite this prior knowledge the landscape of point mutations (or combinations thereof) that cause resistance to asciminib is not well established.

Epistasis, like allosteric regulation, is a well-established evolutionary phenomenon describing the interaction between two or more genotypes that produces a phenotypic effect greater than the sum of their individual contributions. In the context of cancer treatment, the selective pressure exerted by targeted therapies across lines of therapy can drive the sequential emergence of resistance mutations in succession or in combination [22–26]. Here, each successive new generation of drugs takes aim at the resistance liabilities of previous lines of therapy [23, 27, 28]. As a new “next generation” targeted therapy become available, the unknown resistance profile of these new drugs can combine with previous somatic mutations that were selected during frontline therapy to present a combinatorial clinical challenge. Functional genomics now provides a powerful tool to dissect these evolutionary trajectories of drug resistance. Although epistatic mutations are well-documented in the context of antibiotic resistance, particularly concerning β-lactamase [29, 30], intragenic epistasis contributing to drug resistance in cancer targeted therapies remains relatively understudied but is known to occur [31, 32]. Since sequential monotherapy is a common strategy for managing drug resistance in chronic myelogenous leukemia, and ABL1 mutations often drive this resistance, BCR::ABL1 is an valuable model system to investigate the potential for epistatic interactions during the evolution of drug resistance.

This study combined two complementary variant annotation techniques: base editing and deep mutational scanning. We previously showed that these techniques have complementary strengths and weaknesses and can yield data that is quantitatively similar [33]. We aim to use this dual functional genomics approach in a chronic myelogenous leukemia model to systematically identify asciminib resistance mutations, perform the first edit-on-edit epistasis map, and map asciminib resistance measurements with structural detail. Towards this, base editing was initially used to pinpoint potential resistance hotspots, leveraging its ability to introduce A-to-G mutations across the entire length of large endogenous genomic locus. Then, deep mutational scanning, which is capable of introducing all possible missense mutations provided a comprehensive map of resistance mutations within these identified hotspots. The integration of these two techniques is a novel way to utilize the benefits of both methods to overcome the weaknesses of either individual method.

Notably, we discovered novel resistance mutations outside of the kinase domain, including sites in SH3, SH2, and SH2-kinase linker domains. These domains are crucial for asciminib function, as they mediate ABL1 autoinhibition [1, 2]. While resistance to asciminib and prior generations of active site inhibitors has been observed within the kinase domain in clinical settings [16, 34], our findings demonstrate the potential for resistance to also emerge in other ABL1 regions. This work highlights the power of combining base editing and deep mutational scanning to complement each other and identify and characterize these diverse resistance mechanisms.

To further elucidate the mechanisms of asciminib resistance, we also employed a novel ABL1 Förster resonance energy transfer (FRET) biosensor designed to directly measure the conformational state of ABL1 in cells treated with asciminib. Using this biosensor, we demonstrate that asciminib resistance mutations across domains are uniformly associated with a more open ABL1 conformation.

## Methods

### Tissue culture

All cell lines were confirmed to be mycoplasma free (MycoAlert^®^ Mycoplasma Detection Kit, Lonza LT07-710). All cell lines were grown at 37 °C and 5% carbon dioxide atmosphere. HEK293Ts were maintained in DMEM (Corning 10-013-CM) supplemented with 10% FBS (Corning 45000-736) and 1% penicillin streptomycin (Corning 30-002-Cl). Ba/F3s and K562s were maintained in RPMI-1640 (Cytiva SH30027) supplemented with 10% FBS and 1% penicillin streptomycin. Additionally, untransformed Ba/F3s were grown in 1 ng/mL mouse IL-3 (PeproTech 213 − 13).

### Genomic editing of K562s

K562 ABL1 Y253H cells were generated by electroporation (Lonza 4D-Nucleofector X Unit, pulse code FF-120) of 5 million wildtype K562 cells with 5 µg of single guide RNA (sgRNA) (gCCCCGTACTGGCCCCCGCCC) plasmid (Addgene #104991) [35] and 5 µg base editor plasmid ABE8e SpG (Addgene #235044) in Chicabuffer 2M [36]. Following electroporation, cells were allowed to recover in RPMI medium for three days. To select for cells harboring the desired mutations, the electroporated cells were cultured in 1 µM imatinib for two weeks. Genomic DNA was subsequently isolated from the selected cell populations using the Monarch Genomic DNA Purification Kit (NEB #T3010L). The base edited exon was amplified by polymerase chain reaction (PCR) using primers designed with Primer-BLAST [37] and KOD Hot Start Polymerase (Sigma-Aldrich 71842). Finally, Sanger sequencing was performed (Azenta) to confirm the presence of the intended mutations.

### Base editor screen

ABE8e-SpG (Addgene #235044) was generated by deleting the sgRNA expression cassette of pRDA_479 (Addgene #179099) [38]. ABE8e-SpG lentivirus was generated by co-transfecting HEK293T cells with 5 µg of ABE8e-SpG plasmid and an equimolar ratio of helper plasmids (1 µg each) in a 6-well plate using the calcium phosphate transfection method [39]. The day after HEK293Ts were transfected, media was changed to RPMI. The following day ABE8e-SpG lentiviral media was used to infect K562s. Three days after K562 infection, infected cells were selected with puromycin resistance (1 µg/mL). *BCR::ABL1* tiling guide RNA (gRNA) sequences were generated by CHOP-CHOP [40] with ‘NGN’ PAM setting. Guides were cloned into lenti-sgRNA hygro vector (Addgene #104991) [35] by GenScript or golden gate cloning [41]. To generate lentiviral particles encoding the *BCR::ABL1* and control [42] libraries, HEK293T cells were co-transfected with 50 µg of the library plasmids and helper plasmids (10 µg each) in a 10-cm dish using the calcium phosphate method [39]. Two independent biological replicates of K562 cells expressing ABE8e SpG were transduced with sgRNA library lentivirus at a low multiplicity of infection (MOI < 0.3), and a coverage of 500X. Three days post-transduction, cells were selected with 200 µg/mL hygromycin for six days to enrich for cells expressing the sgRNA library.

K562 cells expressing ABE8e SpG and harboring the *BCR::ABL1* sgRNA library (either wild-type or ABL1 Y253H) were treated with DMSO (vehicle control) or kinase inhibitors. Specifically, wild-type (WT) cells were treated with 7.5 nM asciminib for nine days or 40 nM imatinib for 15 days, while ABL1 Y253H cells were treated with 30 nM asciminib for nine days. Following drug treatment, genomic DNA was isolated from each cell population using phenol-chloroform extraction [43]. Quantification of relative sgRNA abundance in each sample was performed by next-generation sequencing as previously described [33, 44]. Reads were trimmed with Cutadapt [45]. Guides were aligned and counted using bowtie [46] and a custom python script, respectively. To ensure that fold-change values were derived from well-quantified endpoints, sgRNAs were only included if their read count across replicates surpassed a threshold of 475 in both the pre- and post-treatment conditions. Differential sgRNA abundance was quantified by calculating log2 fold change (LFC) and Benjamini-Hochberg adjusted p-values using the pyDESeq2 package [47]. The genomic coordinates and functional consequences of the base edits were annotated using the Ensembl Variant Effect Predictor [48], with the final editing location defined by the mean position within the cDNA. To identify likely genotypes from the base editor screen, we utilized BE-Hive [49] under default settings (ABE, mES) to calculate the most probable editing results for each sgRNA.

### Deep mutational scan

*BCR*::*ABL1* saturating mutagenesis libraries of all possible single amino acid changes were synthesized and cloned into the pUltra *BCR*::*ABL1* plasmids (Addgene #210432) by Twist Biosciences. *BCR::ABL1* saturating mutagenesis lentivirus was generated by transfecting HEK293Ts with BCR::ABL1 saturating mutagenesis library (50 µg) and helper plasmids (10:10:10:10 µg) in 10-cm dish using calcium phosphate [39]. Two independent biological replicates of K562s were transduced with *BCR*::*ABL1* saturating mutagenesis lentivirus at low multiplicity of infection (MOI < 0.3) and a coverage of at least 1000X. K562s media was supplemented with 10 µg/mL DEAE-Dextran (ThermoScientific, J63781) to promote transduction [50]. Four days after infection, K562s carrying the BCR::ABL1 saturating mutagenesis library were treated with 125 nM of asciminib for nine days. The asciminib treatment concentration was selected to match the average clinical exposure (after correcting for protein binding) [51] and to account for the increased baseline resistance observed during *BCR::ABL1* cDNA library overexpression (Additional file 1: Fig. S1). Phenol-chloroform was used to purify genomic DNA [43], and Qubit (ThermoScientific, Q32854) was used to quantify the genomic DNA concentration. Mutagenized region-specific next generation sequencing primers were designed by Primer-BLAST [37]. To ensure at least 1000X representation of the library, the required input mass of bulk genomic DNA was calculated based on the transduction efficiency, accounting for the fraction of uninfected cells due to the low MOI. The mutagenized regions were then amplified using a high-fidelity polymerase (NEB Next Ultra II Q5, M0544L) and the following thermocycler program (Table 1).

**Table 1:** PCR program for deep mutational scanning region amplification

Temperature (°C)	Time	Cycles
95	2 min	1
95	10 s	
58	10 s	25
70	5 s	
65	5 min	1

Following PCR product purification (Omega Bio-tek, D6294), 250 bp paired-end sequencing was performed on an Illumina MiSeq system (Azenta, Amplicon-EZ). Variant calling was performed using a custom python script. Briefly, sequences are aligned to *ABL1* cDNA using bowtie2 [52]. If a mismatch appears in both paired ends, then the mismatch is considered a bona fide variant. Insertions, deletion, and compound mutations were ignored. Each mutation must be observed at least 3 times and have a mutant allele frequency greater than 1:4000.

### Growth rate confirmation of hits

Successful base editing and deep mutational scan hits were validated by comparing the growth rates of resistance mutations to controls. Controls included cells transduced with an AAVS1 sgRNA (for base editing hit comparison) and wild-type *BCR*::*ABL1* (for deep mutational scan hit comparison). Cell counts were acquired using a BD Accuri C6 flow cytometer. Exponential growth rate * r* are determined by fitting cell count time course to the following exponential growth model using the CurveFit function in the SciPy package [53].

1 $$\:\begin{array}{c}N={N}_{0}{\:e}^{rt}\end{array}$$

* N* is the final cell count, and * t* is time in terms of hours. The initial count $\:{N}_{0}$ for each independent replicate is allowed to vary ± 10% to allow for error within the count measurement.

Individual growth rates $\:{r}_{i}$ for specific mutant variants were estimated from pooled experiments by measuring changes in their relative frequencies over time. First, the bulk exponential growth rate of the pooled library $\:{r}_{p}$ was calculated using Eq. 1. The specific growth rate for an individual variant was then determined as follows:

2 $$\:\begin{array}{c}{r}_{i}={r}_{p}+\frac{\mathrm{ln}\left({f}_{2}/{f}_{1}\right)}{t}\:\end{array}$$

Where * f* represents the relative frequency of a variant, calculated as the individual read count divided by the total count of the pooled library. The variables $\:{f}_{1}$ and $\:{f}_{2}$ denote the relative frequencies at the initial time point (Day 0) and the final time point (Day 9), respectively, while * t* represents the duration of the growth period between measurements.

To account for potential batch effects between the distinct 8-mer tiles of the saturating mutagenesis library and across experimental replicates, we applied a robust min-max normalization to the growth rates. These batch effects often arise from minor fluctuations in selection pressure, absolute drug concentration, or incubation timing. We specifically chose this normalization approach because deep mutational scan (DMS) screens typically exhibit a bimodal distribution [33, 54–57]; in the context of drug resistance, this is characterized by a large sensitive population and a distinct, smaller resistant population [58]. Consequently, standard Z-score transformations were not applied to the growth rates because they assume a normal distribution. In a bimodal landscape, a Z-score would incorrectly center the data in the low-density valley between the two primary biological states, distorting the functional scores.

To ensure the scaling was resilient to technical artifacts and extreme outliers, such as sequencing noise or lethal variants, we defined the scaling boundaries using the 1st ($\:{P}_{1}$) and 99th ($\:{P}_{99}$) percentiles of the growth rate distribution within each replicate. The normalized growth rate ($\:{P}_{norm}$) was calculated as follows:

3 $$\:\begin{array}{c}{P}_{norm}=\left(\frac{r-{P}_{1}}{{P}_{99}-{P}_{1}}\right)\times\:100\:\end{array}$$

This method effectively implements a null-normalization or basal rescaling strategy, conceptually similar to established frameworks [59–61] for standardizing functional scores in deep mutational scanning. By anchoring the baseline to the 1st percentile, we effectively ‘zero’ the inhibited sensitive population, allowing for a direct comparison of resistance magnitudes across the entire BCR::ABL1 protein landscape. This percentile-based harmonization preserves the biological dynamic range while neutralizing regional differences in library representation and experimental batch effects.

### Cell viability

Cell viability dose-response assays were conducted in 96-well plates. Two thousand K562 cells or five hundred Ba/F3 cells were seeded per well in 100 µL of RPMI medium. Cells were then treated with varying concentrations of the compound using Tecan D300e digital dispenser in triplicate. Three days post-treatment, 100 µL of a 1:5 dilution of CellTiter-Glo 2 (Promega, G9241) reagent was added to each well. Luminescence signals were measured using a PerkinElmer EnVision plate reader. Dose response half maximal concentration $\:{EC}_{50}$ and drug efficacy * Bot* was determined by the following four-parameter formula using the CurveFit function in the SciPy package [53].

4 $$\begin{array}{c}V=\frac{Top-Bot}{1+{\left(\frac{{EC}_{50}}{X}\right)}^{H}}+Bot\end{array}$$

Viability * V* is normalized to DMSO only luminescence signal at different * X* concentration of the compound. The hill slope * H* governs the slope of the curve at the $\:{EC}_{50}$. The * Top* and bottom * Bot* of the sigmoidal are optimized based on the ceiling and floor of the best fit line, respectively. Mean and standard deviation values were reported by rounding the standard deviation to one significant figure and the mean to the same decimal place.

### Epistatic interaction definition

To determine the presence of bona fide epistatic interactions between mutations, we first defined the expected or neutral phenotype of compound mutants under a Product model framework (also known as the Product neutrality function [62]). This model, defined by Mani et al. [63], has been widely used to explore genetic interactions in *Saccharomyces cerevisiae* [62, 64] and antibiotic resistance [65], under the assumption that mutations act independently. Within this framework [63], epistasis is defined as a significant, non-additive deviation of the observed compound phenotype from the theoretical expectation of independence. For any two mutations, * x* and * y*, we define their individual effects * m* relative to the wild-type (* wt*) as $\:{W}_{x}={m}_{x}/{m}_{wt}$ and $\:{W}_{y}={m}_{y}/{m}_{wt}$. The theoretical expected value for the double mutant $\:{m}_{xy}$ is then calculated by multiplying these relative ratios and rescaling by the wild-type value ($\:{m}_{wt}$):

5 $$\:\begin{array}{c}{m}_{xy}={W}_{x}{W}_{y}{m}_{wt}=\left(\frac{{m}_{x}}{{m}_{wt}}\right)\times\:\left(\frac{{m}_{y}}{{m}_{wt}}\right)\times\:{m}_{wt}=\frac{{m}_{x}\times\:{m}_{y}}{{m}_{wt}}\end{array}$$

An epistatic interaction is defined by the difference between the experimentally observed parameter of the compound mutant and this predicted theoretical value ($\:{m}_{xy}$). A significant deviation above the predicted product indicates positive epistasis, allowing us to isolate specific structural or functional interdependencies between residues from simple cumulative effects.

In this study, we applied this framework to $\:{EC}_{50}$ and * Bot* values (Eq. 4) to detect epistatic [63] shifts in drug potency and maximal efficacy [66], respectively. To ensure numerical stability when calculating the Product model [63] for efficacy, a small constant was added to the * Bot* values to prevent division by zero in instances where the lower asymptote approached zero. Furthermore, to visualize the same null model of epistasis across the dose-response landscape, we used an inversion of the dose response curve in Eq. 4 to identify deviations from independence. Specifically, we used an inverted four-parameter sigmoidal curve to map measured viability * V* to its corresponding drug concentration * X* for single mutants:

6 $$\:\begin{array}{c}X=\:{EC}_{50}{\left[\left(\frac{Top-Bot}{V-Bot}\right)-1\right]}^{\frac{1}{H}}\:\end{array}$$

This transformation allowed us to predict the expected shifts in drug potency for the double mutant in the absence of epistasis after applying Eq. 5.

### ABL1 FRET biosensor design, tissue culture, and analysis

A BCR::ABL1 FRET (Addgene #235032) biosensor was developed based on a previously described Src FRET biosensor [67, 68], adapting it for compatibility with the BD Accuri C6 flow cytometer. To prevent fluorophore-induced dimerization, monomeric forms of fluorescent proteins were used. Specifically, mStayGold [69] was inserted into a solvent-exposed loop turn within the SH2 domain of ABL1, and mScarlet3 [70] was inserted at the C-terminus of the kinase domain.

Six hundred thousand HEK293T cells were transfected with 3 µg of the ABL1 FRET plasmid using Lipofectamine 2000 (Invitrogen, 11668027) in a 6-well plate. Transfections were performed in triplicate for each experimental condition. The following day each transfection condition was seeded into a 12-well plate containing DMEM supplemented with either 0.5 µg/mL puromycin and asciminib or DMSO. Media was refreshed the following day and again 24 h later. After a total of four days of treatment, cells were released with trypsin, and at least 5,000 cells from each well were analyzed using a BD Accuri C6 flow cytometer. This incubation period allowed for sufficient expression and maturation of the ABL1 FRET biosensor. Upon excitation with a 488 nm laser, the FRET ratio was defined as the mean fluorescence intensity of a viable cell in the 610 nm channel (mScarlet3 emission) divided by the intensity in the 510 nm channel (mStayGold emission). Flow data was opened with FCSParser [71].

### Statistical analysis and data visualization

Statistical significance of base editor screen enrichment was determined using pyDESeq2 [47]. To confirm base editor screen hits relative to the *AAVS1* control, a Dunnett’s test was performed to correct for multiple comparisons. For the analysis of epistatic interactions, the variance of the theoretical null model was calculated using standard multiplicative error propagation to account for the compounded uncertainties of the single-mutant and wild-type parameters. Curve fitting and parameter estimation were performed using the SciPy optimization package [53]. To evaluate deviations between the observed double-mutant phenotypes and theoretical null predictions, we employed Welch’s t-test. This test was selected to account for unequal variances, as the null model variance was derived from the propagation of three independent uncertainties whereas the observed double-mutant data represented a single experimental distribution. AlphaFold3 [72] was used to generate ABL1 and SHP2 structures. Structural visualizations were generated using PyMOL [73], and phenotypic data were visualized using the Seaborn library [74]. All replicates (*N*) represent independent biological replicates.

## Results

### A protein-wide search for asciminib resistance maps hotspots in and out of the kinase domain

**Fig. 1:**
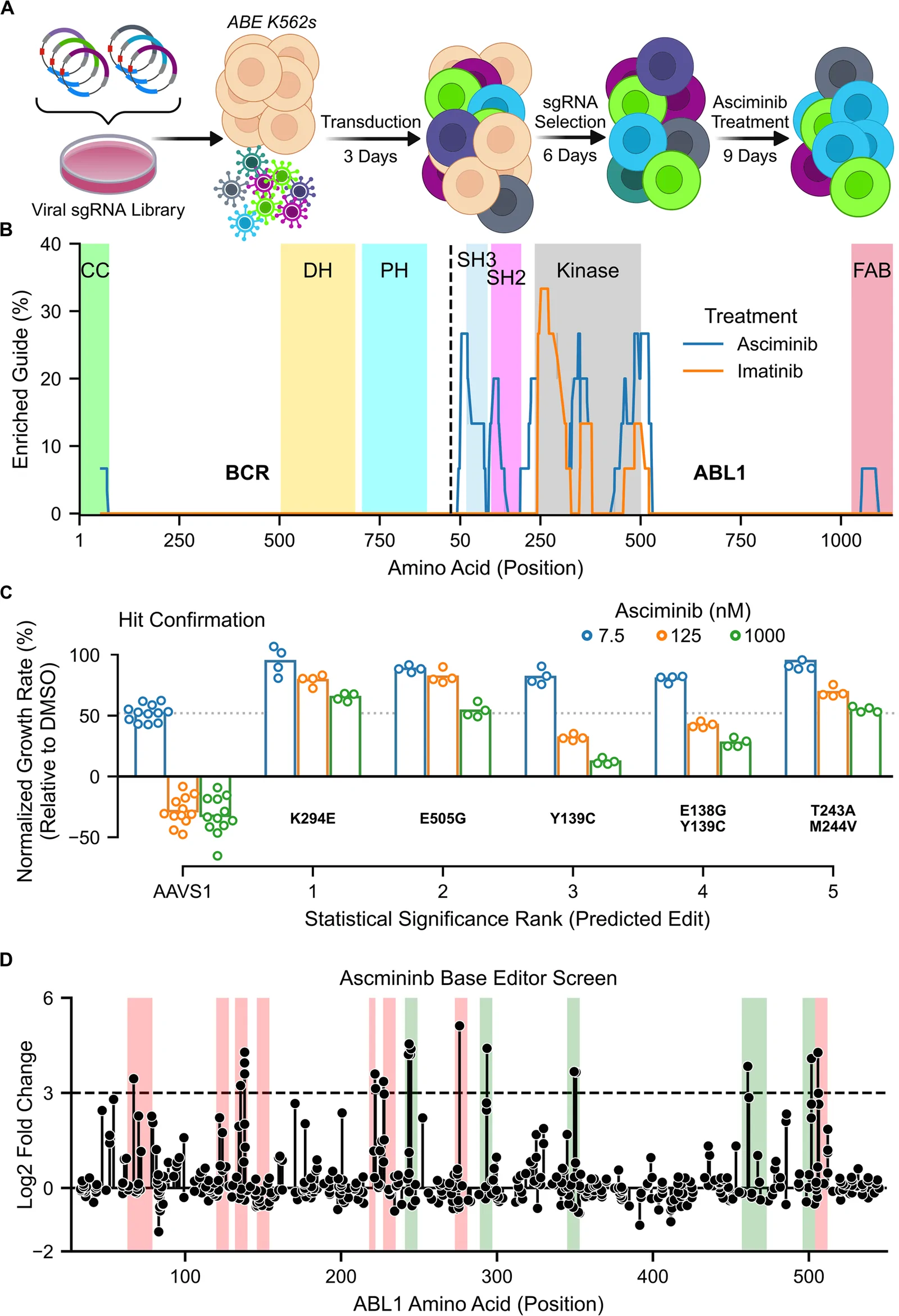
Base editor screen identifies asciminib resistance in ABL1 SH3, SH2, and kinase domains. **A** ABE screen timeline. K562 cells were transduced with an ABE library targeting *BCR::ABL1* and subjected to 9 days of asciminib selection. Cell pellets were collected immediately before and after asciminb treatment for genomic DNA extraction and next-generation sequencing of sgRNA abundance. (*N* = 2). **B** Rolling relative frequency of enriched sgRNAs across the BCR::ABL1 protein. The y-axis represents the percentage of guides within a sliding window that meet the significance thresholds (log2 fold change > 1.25 and adjusted *p* < 0.01). BCR::ABL1 domains are labeled as follows: coiled-coil (CC), Dbl-homology (DH), Pleckstrin-homology (PH), Src-homology 3 (SH3), Src-homology 2 (SH2), kinase, and F-actin binding domain (FAB). **C** Functional confirmation of top asciminib resistance hits. K562 cells expressing ABE and individual candidate sgRNAs were treated with 7.5, 125, or 1000 nM asciminib. Base editing predictions were generated using BE-Hive [49] to identify the most probable amino acid substitutions. All candidates maintained significant enrichment relative to the AAVS1 control at all doses (Dunnett’s test *p* < 0.001), demonstrating that these hits confer high-level resistance even at supraphysiological concentrations. (*N* ≥ 4). **D** Lollipop plot showing the location of potential asciminib resistance mutations identified by adenosine base editing screen across ABL1 protein. Dashed line represents the log2 fold change + 3. Red regions indicate hotspots of novel resistance (e.g. L69P, Y139C, D276G, E505G), green regions represent locations of previously reported resistance mutations (e.g. M244V, K294E, M351T, V468F, and I502). These regions were selected for follow up by deep mutational scanning. (*N* = 2)

To systematically investigate the existence of asciminib resistance mutations across the entire BCR::ABL1 protein, we employed an adenosine base editor screen with 1555 sgRNAs tiling across the entire *BCR*::*ABL1* gene in K562 cells (Fig. 1A) [75]. We also performed a parallel resistance screen with imatinib as a positive control. To maximize the diversity of identified resistance mutations, we selected drug concentrations near the *EC*_*50*_, providing a permissive selective pressure that captures both moderate and high-level resistance alleles. The strong replicate correlation in the primary screen (Additional file 1: Fig. S2A, S2B; Additional file 2) supports the robustness of our screening results. Dramatically expanding on previous findings, we systematically identified sgRNAs targeting the ABL1 SH3, SH2, and kinase domains that displayed significant enrichment following asciminib treatment (Fig. 1B, S2C). In contrast, K562 cells treated with imatinib identified sgRNA enrichment predominantly in the kinase domain (Additional file 1: Fig. S2D), consistent with prior work in Ba/F3s [10, 76]. These findings are consistent with the distinct mechanisms of action of each inhibitor and the well-established clinical resistance profile of imatinib [24, 77]. Notably, all eight of the most potent imatinib resistance hits (LFC > 1.25, p-adj < 0.01) also conferred significant resistance to asciminib, indicating a shared vulnerability between the two binding sites. This is in contrast to initial reports on the resistance spectrum by Wylie et al. [18] but consistent with recent reports of F359X mutations causing clinical resistance against nilotinib and asciminib [19].

To validate the reliability of the primary screen, we functionally assessed the five most statistically significant enriched sgRNAs as single constructs [75]. These guides targeted known asciminib resistance mutations like K294E and M244V in the kinase domain [16, 18] alongside novel mutations, including Y139C in the SH2 domain and E505G in the myristate binding site. All five candidate guides, each exhibiting an LFC > 3 in the primary screen, successfully replicated the resistance phenotype (Fig. 1C; Dunnett’s multiple comparison to AAVS1 control, *p* < 0.001). Strikingly, these sgRNAs maintained positive growth at 125 nM and 1000 nM asciminib, with the latter concentration significantly surpassing the clinically relevant unbound plasma concentration [78].

While the initial base editing screen utilized a low selective dose of 7.5 nM asciminib to maximize hit sensitivity and discovery, we prioritized hotspots with an LFC > 3 and an adjusted *p* < 0.001 to identify the most clinically significant resistance sites. This strategy identified six novel hotspots alongside known resistance regions used as positive controls (Fig. 1D). Although base editing phenotypes correlate strongly with deep mutational scanning (DMS), the technology is constrained by A-to-G activity and susceptible to confounding bystander edits [33]. To resolve these limitations and capture non-editable resistance mutations, we followed up on all identified hotspots using DMS (Fig. 1D). This higher resolution approach provides the structural granularity necessary to elucidate the mechanisms underlying these novel asciminib resistance hotspots.

### Saturation mutagenesis provides structural insights in base editing hotspots

Following our base-editing screen, we employed deep mutational scanning (DMS) to evaluate a comprehensive library of all 20 amino acid substitutions at each residue of interest [79]. We have previously validated that heterologous expression of the cDNA and endogenous editing yield quantitatively similar results in BaF3 and K562 cells [33]. This focus on BCR::ABL1 structural variants is supported by the exceptional selectivity of asciminib [11, 18]; unlike active-site inhibitors that target multiple kinases, asciminib only significantly inhibits *ABL1*-driven leukemias (*IC*_*50*_ < 2 µM) when screened across 495 cancer cell lines [80]. Leveraging this specificity, we targeted the key resistance regions identified in our initial base-editing screen for DMS analysis. We hypothesized that the greater mutational diversity of DMS would reveal additional resistance variants missed by the inherent constraints of base editing, providing deeper structure-function insights.

**Fig. 2:**
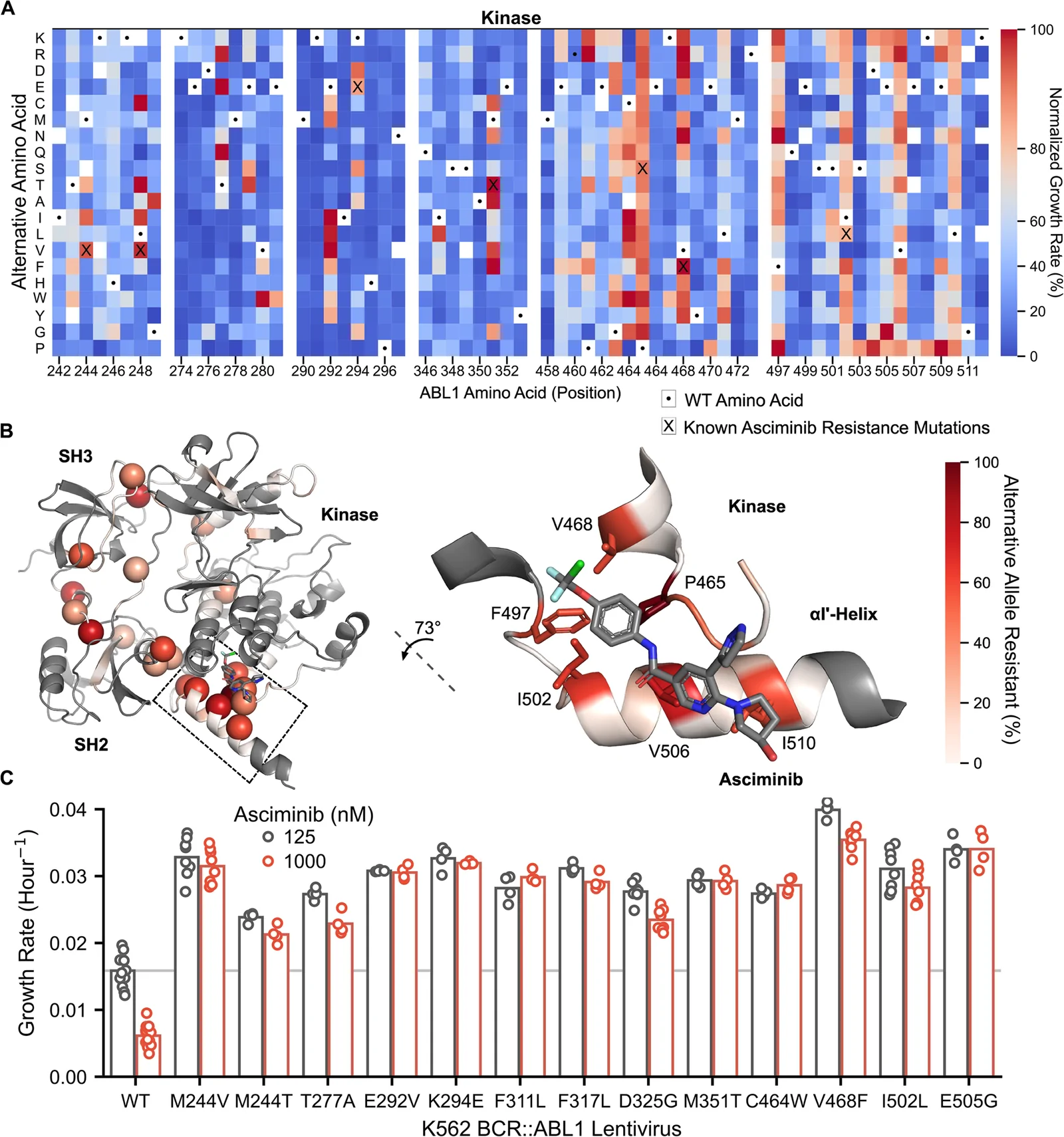
Asciminib resistance hotspots in ABL1 kinase domain. **A** Heatmap of normalized mutant growth rates following a deep mutational scan of resistance hotspots in the ABL1 kinase domain. K562 cells overexpressing BCR::ABL1 were treated with 125 nM asciminib for 9 days. Red indicates higher growth rate, and blue indicates lower growth rate. Dots (•) represent wild-type (WT) residues, and ‘X’s indicates known asciminb resistance mutations. Empty white cells represent data points excluded due to low confidence. (*N* = 2). **B** Structural mapping of resistance hotspots. Left: ABL1 structure (AlphaFold3/PDB 5MO4) is color-coded by the percentage of variants at each position with a normalized growth rate > 80%. The dashed rectangle indicates the region shown in the close-up view on the right. Right: Enlarged view of the myristoyl binding pocket, rotated 73° relative to the initial structure. Residues with > 25% highly resistant substitutions (> 80% normalized growth rate) are shown as spheres. Color intensity reflects the percentage of resistant variants per position, highlighting hotspots such as the αI’-helix and residues V468, P465, and F497. **C** Growth rates of K562 overexpressing BCR::ABL1 mutants treated with asciminib. Solid horizontal lined represents the *WT* BCR::ABL1 growth rate treated with 125 nM asciminib. (*N* ≥ 4)

We conducted the follow on DMS screen at a higher asciminib concentration (125 nM) compared to the original base editor screen (7.5 nM) [79]. The chosen asciminib concentration approximates the average effective concentration in the clinic after accounting for serum binding [51, 81, 82]. Independent replicate experiments demonstrated a strong Pearson correlation of 0.92 of the DMS measurements, indicating high assay reproducibility (Additional file 1: Fig. S3A; Additional file 3). To define resistance, we adopted a threshold of a normalized growth rate of > 80% as a stringent cutoff for defining strong resistance (Additional file 1: Fig. S3B). The validity of our resistance cutoff is supported by the observation that clinically reported resistance mutations, such as M244V, M351T, V468F, and I502L [16, 34, 83], that exhibit strong resistance and meet this threshold (Fig. 2A). To rule out stability artifacts, we confirmed via dot blot (Additional file 1: Fig. S3C) that representative hits maintain expression levels comparable to *WT* (Dunnett’s test, *p* > 0.01). Beyond known residues, we identified 279 novel asciminib resistance variants with normalized growth rates greater than 80%.

As expected, we observed significant resistance within the αI’-Helix in the myristate pocket. Beyond a few previously reported substitutions at these sites, we found that nearly all amino acid variants at positions F497, I502, V506, and L510 conferred potent resistance (Fig. 2B). Even when the substitution patterns are biophysically conservative we observe surprising phenotypes. This includes the observation that V506L and V506T confer potent asciminib resistance but V506I does not. The emergence of a ‘proline resistance band’ is in stark contrast to proline bands observed in other studies where proline substitution is largely deleterious. An αI-Helix kink induced by proline underscores the importance of maintaining secondary structure and the non-polar core of the myristate binding site for asciminib inhibition. A particularly potent and novel resistance mutation, E505G, identified by both base editing and DMS screens, further highlights the significance of this region (Fig. 2C). Similarly, beyond this key binding pocket, other kinase domain asciminib resistance mutations (K294E, D276G) were also identified through our combination of DMS and base editing.

**Fig. 3:**
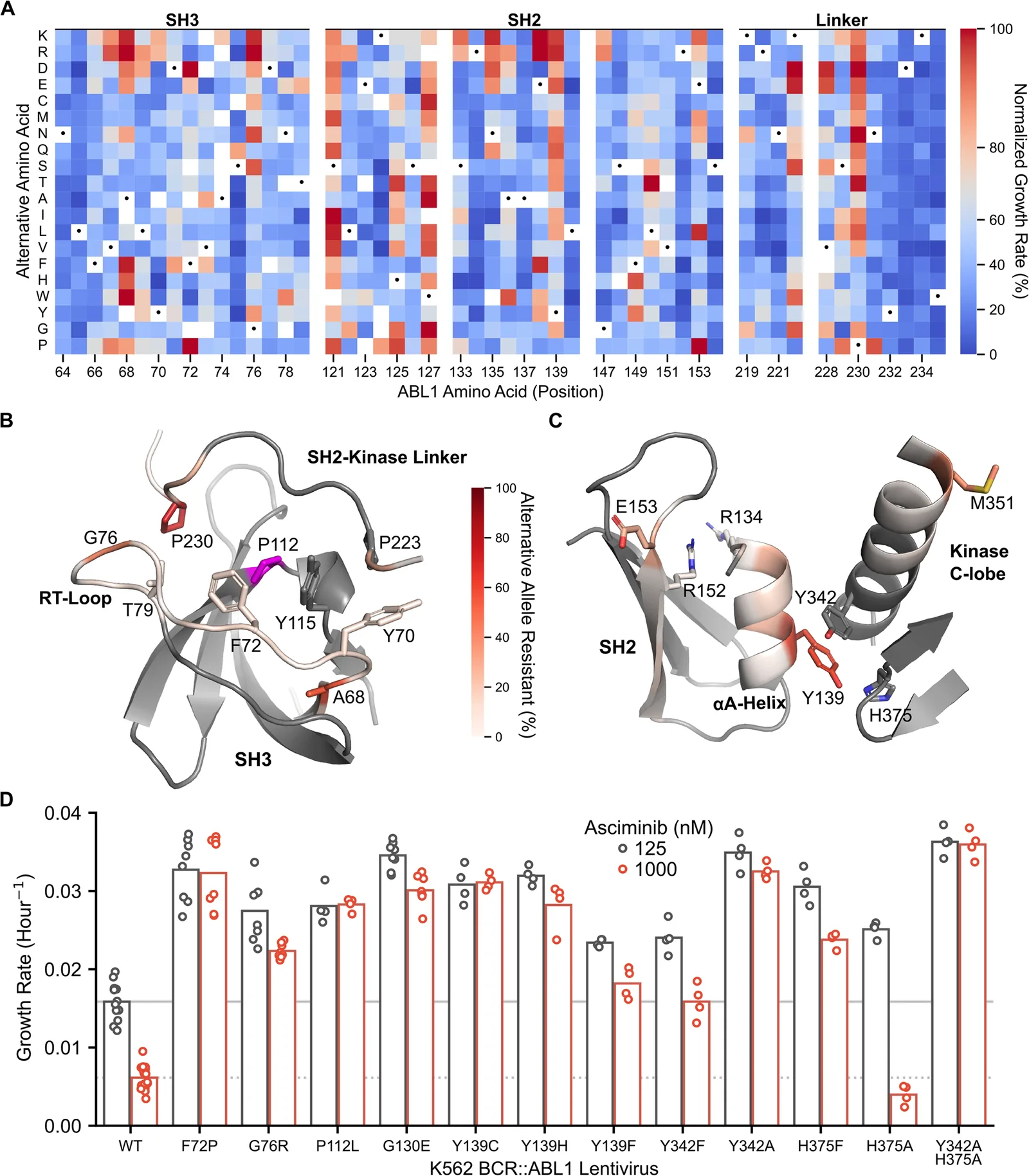
Asciminib resistance hotspots in ABL1 regulatory domains. **A** Heatmap of normalized mutant growth rates following a deep mutational scan of resistance hotspots in ABL1 regulatory domains. K562 cells overexpressing BCR::ABL1 were treated with 125 nM asciminib for 9 days. Red indicates higher growth rate, and blue indicates lower growth rate. Dots (•) represent wild-type (WT) residues. Empty white cells represent data points excluded due to low confidence. (*N* = 2). Percentage of mutants with a normalized growth rate greater than 80% mapped onto the ABL1 **B** SH3 domain, and **C** SH2 domain. Deeper red indicates a higher percentage of variants at that position that are highly resistant (**B**, **C**). (PDB 5MO4, ABL1 AlphaFold3). **D** Growth rates of K562 overexpressing BCR::ABL1 mutants treated with asciminib. Solid and dotted horizontal lines indicate the *WT* BCR::ABL1 growth rate treated with 125 and 1000 nM asciminib, respectively. (*N* ≥ 4)

Beyond the kinase domain, our DMS analysis identified potent asciminib resistance mutations within the regulatory ABL1 SH3 and SH2 domains (Fig. 3) [84]. While previous deletion and tool molecule studies had implicated these regions in resistance to myristate pocket inhibitors [11, 20, 21, 85], our screen is the first to identify multiple specific point mutations that confer resistance to a clinically approved drug. We discovered that mutations in the region linking the SH2 domain to the kinase domain, particularly at P230, conferred potent resistance (Fig. 3A). This result highlights the critical role of the autoinhibitory SH3-linker interaction in determining asciminib efficacy. Although not a canonical PXXP motif, the SH2-kinase linker’s prolines P223 and P230 achieve a remarkable fit within sites 1 and 3 of the ABL1 SH3 domain [86] (Fig. 3B). Prior studies have shown that point mutations of these specific prolines are sufficient to increase ABL1 kinase activity and induce cellular transformation [1, 4, 87–89]. Our data now demonstrates that this known activation mechanism directly translates to potent asciminib resistance by abrogating the intramolecular interaction between the SH3 domain and the SH2-kinase linker. This mechanism was further supported by two additional lines of evidence from our study. First, mutations within the SH3 domain’s RT loop, which forms the binding surface, also caused resistance (Fig. 3A and B, and 3D). Second, introducing the well-characterized SH3 loss-of-function mutation [4, 88–91], P112L, was sufficient to induce high-level asciminib resistance (Fig. 3D). These independent lines of evidence confirm that the functional integrity of the SH3 domain is essential for asciminib’s mechanism of action [4, 91].

We also gained a new insight into the structure-function relationships in the SH2 domain. The SH2 domain’s phosphotyrosine binding pocket is partially formed by the αA-Helix and canonically relies on key residues like R152, located within the conserved FLVR motif, to provide electrostatic interactions with the negatively charged phosphate group of a phosphotyrosine [92–94]. No R152 mutations were found to confer resistance to asciminib (Fig. 3A), suggesting that the canonical phosphotyrosine binding function of the SH2 domain function is dispensable for drug resistance. In contrast, another αA-Helix residue, Y139, which points away from the phosphotyrosine pocket toward the C-lobe of the ABL1 kinase, has emerged as a key player in drug resistance (Fig. 3C). Tyrosine 139 is positioned to form π-π stacking interactions with Y342 [95] and hydrogen bonding with H375 (Fig. 3C and S3D). Base editing, DMS, and overexpression experiments implicated Y139C/H mutations in conferring resistance (Fig. 3A and D). These mutations likely disrupt the allosteric SH2-kinase interaction, leading to increased ABL activity and resistance to asciminib. If this interaction was responsible for maintaining a closed ABL1 conformation we would expect mutations at Y139, Y342, and H375 to all cause resistance. Multiple independent mutations at all three of these sites support this resistance hypothesis (Fig. 3D). In sum, while mutations in the SH2 domain can cause drug resistance, the canonical phosphotyrosine binding function is dispensable, while a proposed tripartite interaction between residues Y139, Y342, and H375 appears to be required for autoinhibition and asciminib activity.

Finally, we wanted to examine the ability of these mutations to exhibit high level resistance at concentrations as high as 1000 nM (Fig. 2C, and 3D). Surprisingly, most mutations we discovered generate significant resistance at 1000 nM. This indicates the potential utility of these findings to the clinic.

### ABL1 Y253H and V73A cause epistatic resistance to asciminib

Given that single residue mutations can disrupt ABL1 autoinhibition via the SH3 or SH2 domains (Fig. 3A), conferring resistance to asciminib, and the prevalence of mutations in relapse/refractory CML patients, we hypothesized that certain combinations of mutations might exhibit epistatic interactions, leading to resistance to asciminib. This phenomenon, whereby the combined effect of two mutations is greater than the sum of their individual effects, is well-established in genetics [29, 30, 62–65, 96] and has been observed in the context of tyrosine kinase inhibitor resistance. For example, the E255V and T315I mutations confer significant resistance to ponatinib, but only when present together [32].

**Fig. 4:**
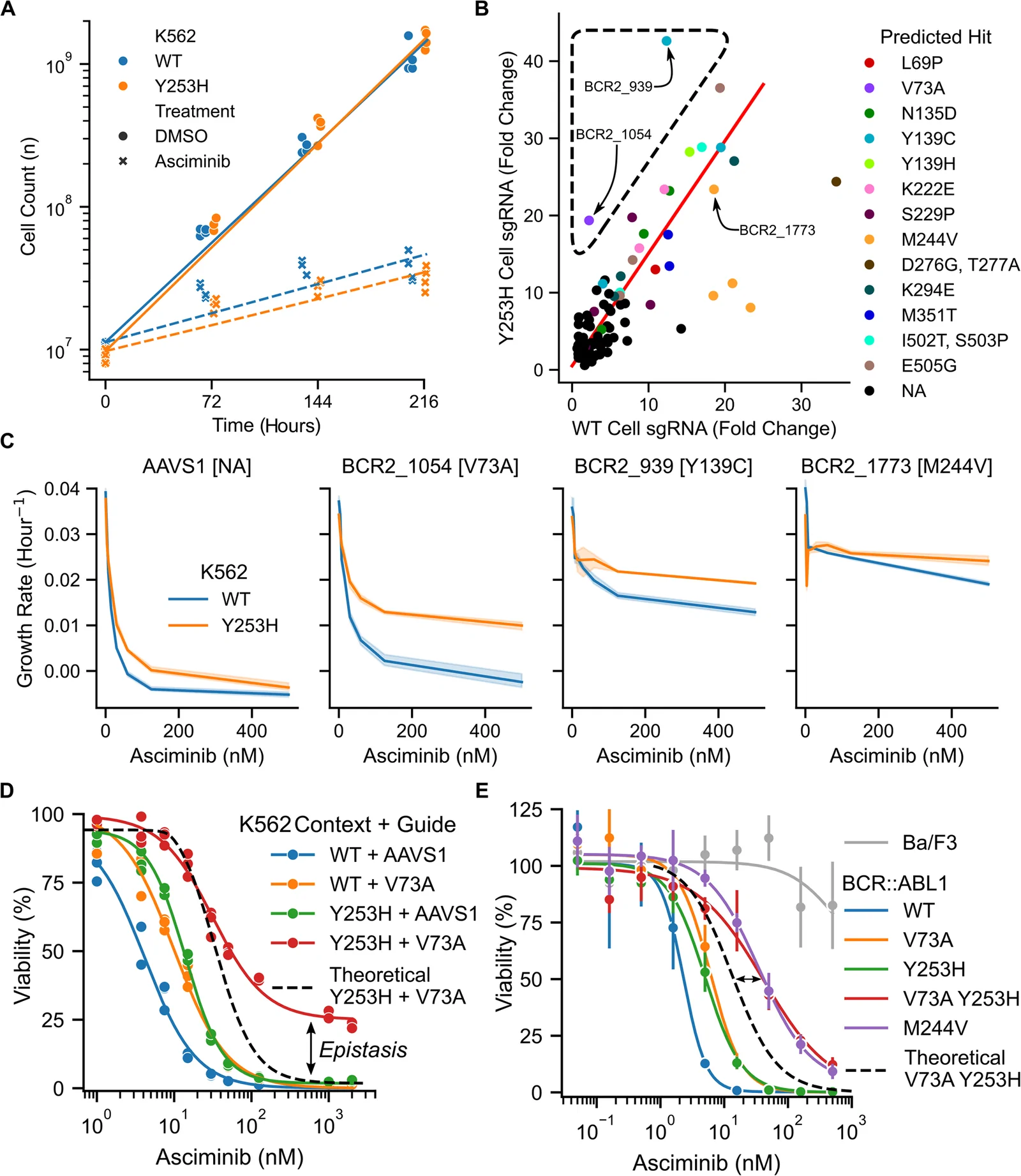
Epistatic asciminib resistance conferred by ABL1 V73A and Y253H. **A** Modeled cell growth curves from the *BCR::ABL1* base editor screen in WT and Y253H K562 cells treated with asciminib or DMSO control. (*N* = 4). **B** Comparison of asciminib resistance conferred by guides in WT and Y253H K562 cells from the base editor screen. Each point represents a guide, color-coded by the predicted resistance ABL1 amino acid edits within the editing window. Guides with a fold change > 1.5 and adjusted *p* < 0.01 in either background were considered for analysis. Red line indicates robust linear regression. The dashed boarder indicates sgRNAs with greater resistance to asciminib in context of Y253H. (*N* = 2). **C** Growth rates of adenosine base-edited K562 cells expressing asciminib resistance-conferring guides and treated with varying concentrations of asciminib. (*N* = 3). **D** Dose-response curves of asciminib in WT and Y253H adenosine base-edited K562 cells expressing control (AAVS1) or V73A-targeting guides. The dashed curve represents the predicted response for the V73A + Y253H double mutant under a Product model of genetic interaction [63], assuming an absence of genetic epistasis (Eqs. 5 and 6). (*N* = 3). **E** Dose-response curves of asciminib in Ba/F3 cells expressing various BCR::ABL1 mutants. The dashed curve represents the predicted response for the V73A Y253H double mutant under a Product model of genetic interaction [63], assuming an absence of genetic epistasis (Eqs. 5 and 6). (*N* = 3)

Given imatinib’s high efficacy and generic availability, it is likely to remain a first-line treatment option for some patients, even with the recent approval of asciminib in the frontline setting [97, 98]. One of the most common resistance mutations to imatinib is the P-loop mutation Y253H [28, 99], which can be readily generated using a adenosine base editor due to its A > G nucleotide change (Additional file 1: Fig. S4A). Moreover, the Y253H position in the P-loop of the N-lobe of the ABL1 kinase was a particularly attractive place to look for epistasis because it is distant from the myristoyl binding pocket, and the nearby mutation M244V is a known single mutant that confers high level imatinib [100] and asciminib [16] resistance. We successfully generated K562 cells with the endogenous ABL1 Y253H mutation (Additional file 1: Fig. S4D). As a control we also generated K562 ABL1 T315I cells (Additional file 1: Fig. S4B, S4E). As expected, these cells were highly resistant to imatinib but remained sensitive to asciminib and ponatinib (Table 2). Importantly, K562 Y253H cells exhibited a slight increase in resistance to asciminib compared to wild-type cells. This difference exists in vitro, but is not clinically relevant because Y253H is not contraindicated in the clinic [83]. To account for this baseline difference, we adjusted the asciminib dosage during the base editor screen to 30 nM, allowing for a direct comparison between *WT* and Y253H K562 base editor screens at about 30% of DMSO treated growth rate (Fig. 4A) [101].

**Table 2:** Dose response *EC*_50_ values of base edited imatinib resistance mutations in K562s (*N  = 3)*

K562	Imatinib (nM)	Ponatinib (nM)	Asciminib (nM)
WT	75	0.17	9.1
Y253H	> 1000	0.59	12
T315I	> 1000	0.56	21

Most resistance-conferring guides (fold change > 1.5, adjusted *p* < 0.01) showed a robust positive association (*p* < 0.001) in their effects between the WT and Y253H K562 cell screens, suggesting that epistasis is rare here. However, two notable exceptions were observed: guides targeting SH2 Y139C (BCR2_939) and SH3 V73A (BCR2_1054) (Fig. 4B). These two guides were the most differentially enriched when comparing the endpoints of the *WT* and Y253H screens (Additional file 1: Fig. S4G, Supplementary File 1) [101]. Notably, the V73A and Y139C guides exhibited a disproportionate increase in resistance when introduced in the Y253H background compared to the WT background, outperforming the M244V control in the mutant context (Fig. 4C) [101]. While the V73A guide showed near-wild-type sensitivity alone, its combination with Y253H dramatically increased the dose-response lower asymptote or *Bot* parameter (Eq. 4) (Fig. 4D). Under the Mani et al. Product model (Eq. 5) [63], the predicted *Bot* parameter for the V73A Y253H double mutant was 3% of relative viability. However, our measured *Bot* parameter for V73A Y253H was 25% relative viability (Additional file 1: Table S1). This significant divergence (Welch’s t-test, *p* < 0.001) confirms a positive epistatic interaction that specifically compromises drug efficacy [66]. This suggests that these two residues are functionally interdependent, allowing the protein to maintain an active state even under high concentrations of asciminib.

To rule out the possibility that off-target or bystander base editing might contribute to the observed resistance, we introduced mutant *BCR::ABL1* into Ba/F3 cells via lentiviral transduction. In this model, where 100% of the population carries the specified variants, epistasis is reflected by a distinct shift in the *EC*_*50*_ of the dose-response curve (Eq. 4). Similar to our results in K562 cells, the *EC*_*50*_ values for V73A and Y253H as single mutants were comparable. Under the Mani et al. Product model (Eq. 5) [63], the predicted *EC*_*50*_ for the V73A Y253H double mutant was 13 nM. However, our measured *EC*_*50*_ parameter for V73A Y253H was 40 nM (Additional file 1: Table S2). This significant divergence (Welch’s t-test, *p* < 0.001) confirms a positive epistatic interaction that compromises asciminib potency [66]. Notably, the V73A Y253H double mutant exhibited a level of resistance comparable to the clinically relevant M244V variant (Fig. 4E) [101].

Interestingly, while the K562 cell line showed increased basal resistance, this phenotype was not observed in the Ba/F3 cells expressing the V73A Y253H double mutant. Given that V73 is primarily solvent-exposed and lacks obvious direct interactions with the kinase domain, the mechanism by which V73A, and its positive epistatic interaction with Y253H, contributes to ABL1 conformational changes and resistance remains an open question.

### A single biophysical measurement in BCR::ABL1 is highly correlated with asciminib sensitivity in cells

**Fig. 5:**
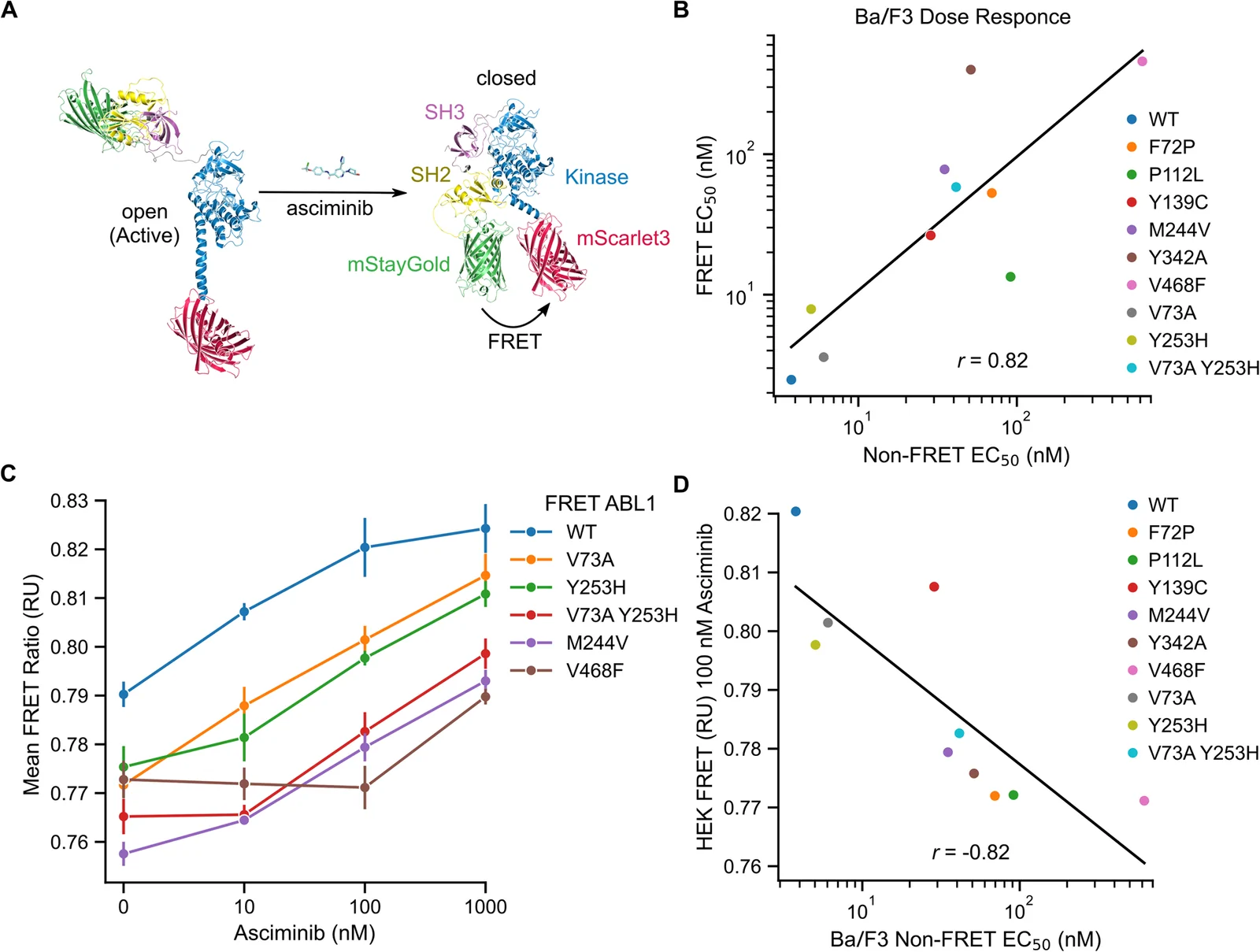
ABL1 FRET biosensor corroborates functional resistance. **A** Schematic representation of the ABL1 FRET sensor and asciminib-induced conformational changes. BCR and flanking ABL1 structures are omitted for clarity. The sensor consists of mStayGold inserted into a solvent-accessible loop within the SH2 domain and mScarlet3 inserted immediately following the kinase domain. In the open (active) conformation, the fluorophores are spatially separated, resulting in low Förster resonance energy transfer (FRET). Asciminib binding stabilizes the closed (inactive) conformation, bringing the fluorophores into close proximity and increasing FRET. Structures were generated using AlphaFold3 [72]. **B** Comparison of asciminib *EC*_50_ values in Ba/F3 cells expressing unmodified vs. FRET biosensor-inserted BCR::ABL1 variants. (*N* = 3). **C** Asciminib dose response analysis of ABL1 FRET sensor activity in HEK293T cells. Cells expressing the ABL1 FRET biosensor were treated with increasing concentrations of asciminib, and FRET ratios were determined by flow cytometry, measuring the ratio of mScarlet to mStayGold emission upon stimulation with 488 nm blue laser. (*N* = 3). **D** Comparison of growth inhibition and drug-induced FRET shifts. Pearson correlation between asciminib *EC*_50_ values in Ba/F3 cells expressing BCR::ABL1 and mean FRET ratios in HEK293T cells expressing the BCR::ABL1 FRET biosensor treated with 100 nM asciminib. (*N* = 3)

To elucidate the mechanism underlying the epistasis between V73A and Y253H, and to understand asciminib resistance more broadly, we investigated the impact of these mutations on ABL1 kinase conformations [102–104]. To monitor ABL1 conformation, we generated a novel BCR::ABL1 FRET biosensor based on a previously validated Src FRET biosensor [67, 68]. This sensor incorporates mStayGold [69] donor fluorophore within a solvent accessible loop turn in the SH2 domain and mScarlet3 [70] acceptor fluorophore immediately following the kinase domain (Fig. 5A, S5). In the closed (inactive) conformation, the fluorophores are in close proximity, resulting in high FRET. In the open (active) conformation, the fluorophores are spatially separated, leading to low FRET [1–3, 102, 103]. To confirm that our BCR::ABL1 biosensor design accurately detects structural transitions, we engineered an analogous sensor for SHP2, a phosphatase governed by similar open-to-closed conformational dynamics [105]. In this parallel model, we demonstrated that a known activating mutation [106, 107] promotes the open (active) state, while an allosteric inhibitor stabilizes the closed (inactive) state [105, 108, 109]. This successful recapitulation of the SHP2 regulatory mechanism [105], which mirrors the conformational dynamics of BCR::ABL1 in response to asciminib, confirms the robustness and generalizability of our biosensor platform (Additional file 1: Fig. S6).

To ensure the BCR::ABL1 FRET biosensor is a biologically faithful reporter, we validated its function across three essential parameters using the Ba/F3 cell model: We confirmed that the catalytic activity required for cellular transformation remains preserved, as the biosensor construct successfully induced growth factor-independent proliferation (Fig. 5B). We further established that the myristoyl binding pocket and allosteric regulatory machinery are functionally intact, evidenced by the construct’s maintained sensitivity to asciminib (Fig. 5C). Finally, we demonstrated that the native pharmacological profile remains undistorted by the fluorophore insertions, which is reflected in the strong Pearson correlation (*r* = 0.82) in cellular drug sensitivity observed across a panel of resistance mutations in both the FRET-modified and unmodified backgrounds (Fig. 5B) [104].

To assess the impact of asciminib resistance mutations on ABL1 conformation, we transfected HEK293T cells with a BCR::ABL1 FRET biosensor incorporating resistance mutations and treated the cells with asciminib. As expected, increasing asciminib concentrations induced a dose-dependent increase in the FRET Ratio in cells expressing the *WT* BCR::ABL1 FRET biosensor, reflecting a shift towards the closed conformation as asciminib binds (Fig. 4C). To confirm that this signal reports specifically on intramolecular movement, we performed a negative control using separately labeled fluorescent BCR::ABL1 proteins; the absence of a FRET increase in this condition (Additional file 1: Fig. S5C) confirms that the sensor detects the transition between the known open and closed conformations of BCR::ABL1 rather than intermolecular artifacts [102, 103, 110]. In contrast, known resistance mutations [14–16, 83] in the myristoyl binding pocket (V468F) and the N-lobe (M244V) exhibited reduced sensitivity to asciminib-induced conformational changes, effectively remaining in a drug-resistant “open” state.

To investigate the mechanism of epistasis between the V73A and Y253H mutations, we introduced the corresponding single and the double mutant into the FRET construct. Consistent with the low level of asciminib resistance observed in both K562 and Ba/F3 cell lines, the V73A and Y253H single mutants exhibited similar and strong dose-dependent FRET ratios that reflect asciminib binding and closing the protein conformation [104]. This mirrored, but did not quite reach the high FRET ratios observed in the *WT* protein. The V73A Y253H double mutant displayed a FRET ratio that appeared similar to the highly resistant M244V variant [16]. This FRET ratio similarity aligned with the observed resistance phenotype in viability assays of the V73A Y253H and M244V in Ba/F3 cells (Fig. 5C) [104]. Taken together, the FRET profile of the V73A Y253H double mutant indicates a failure of asciminib-induced conformational clamping relative to the single mutants, resulting in a structural state indistinguishable from the high-resistance M244V mutant. Notably, we did not apply the Product model of genetic interaction [63] to this dataset, as it remains undefined whether non-linear biophysical readouts [111, 112] (such as FRET efficiency [113]) adhere to the same multiplicative scaling laws governing fitness phenotypes [62, 96].

The biophysical convergence between the V73A Y253H double mutant and the M244V and V468F single mutants is notable, as it suggests that mutations at disparate structural sites can yield nearly identical conformational phenotypes. To investigate how much asciminib resistance in cells can be explained by this simple biophysical switch between an open and closed state, we performed a quantitative comparison between our mutant viability data and our FRET biosensor measurements at 100 nM. Remarkably, we observed a strong negative Pearson correlation (*r* = -0.82) between the concentration-response *EC*_*50*_ values and their corresponding FRET ratios (Fig. 5D) [104]. This relationship establishes the FRET assay as a robust measure of cellular target engagement and strongly suggests that resistance is rooted in diminished on-target engagement and a failure to induce the requisite active-to-inactive ABL1 conformational transition. To our knowledge, this provides the first direct evidence in live cells linking the failure of an allosteric conformational shift to the magnitude of clinical drug resistance.

## Discussion

Combining the breadth of base editing, which can rapidly introduce mutations throughout a large protein, with the depth of deep mutational scanning, which generates all possible missense mutations, this study maps asciminib resistance hotspots, revealing key sites and structural details driving drug resistance. Unlike imatinib, where resistance primarily arises from kinase domain mutations [10, 114], we observed asciminib resistance mutations distributed across the SH3, SH2, linker, and kinase domains. This broader distribution highlights a key difference in mechanisms of action between these two inhibitors. Wylie et al. [18] initially suggested that active-site and myristate-pocket inhibitors possess completely non-overlapping resistance profiles, a finding supported by their study of barcoded KCL-22 cells. Furthermore, Wylie et al. [18] demonstrated that while combination therapy with nilotinib and asciminib successfully suppressed the emergence of resistance, sequential therapy failed to do so. However, this barcoding approach relied on the native genetic diversity present in the parental cell line without mutagenesis, limiting the screen to only those resistance mechanisms already present in the starting population. In contrast, our systematic mutagenesis screens introduce mutations across *BCR*::*ABL1* to comprehensively map resistance, including rare mutations that can emerge under clinical selection pressure but may be absent from un-mutagenized cell lines.

Clinical evidence has now revealed cross-resistance not captured in the Wylie et al. [18] model. Eide et al. [19] identified F359V/C/I mutations in CML patients that confer resistance to both active-site inhibitors (imatinib, nilotinib) and asciminib. These mutations are now recognized in Journal of the National Comprehensive Cancer Network guidelines [115] as contraindicated for asciminib use. Consistent with these clinical observations, our base editor screen identified 8 sgRNAs conferring cross-resistance to both imatinib and asciminib (Fig. 1B). While this overlap represents a minority of resistance mechanisms, it indicates that the resistance profiles are not completely independent as initially suggested by Wylie et al. [18]. This raises concerns about ongoing clinical trials combining imatinib and asciminib [116, 117], as cross-resistant mutations like F359X would render both drugs ineffective. We emphasize that combination therapy remains a powerful strategy for preventing resistance when drugs have truly non-overlapping profiles. Our findings simply indicate that the specific pairing of imatinib and asciminib has more overlap than initially appreciated, which should inform rational combination design and clinical monitoring strategies.

Our deep mutational scanning data strongly corroborates previously reported asciminib resistance mutations within the kinase domain, including M244V, M351T, V468F, and I502L [12, 14–16]. We also identified novel αI’-helix level resistance mutations at E505, V506, and L510 that we expect to eventually arise in the clinic. Beyond the kinase domain, we richly elucidate extensive resistance across the SH3, SH2 and linker domains. Given that SH3 domain deletion confers asciminib resistance [20, 21], it is perhaps unsurprising that we also observed resistance mutations within this domain and that these mutations appear in positions that are known to ablate canonical SH3 function. While SH3 resistance appears driven by the ablation of canonical function, our analysis of the SH2 domain reveals a more complex, non-canonical structural mechanism. We demonstrate that while the conserved arginine residue that is responsible for phosphotyrosine binding [87–89] in SH2 domains has no role in drug resistance, a putative tripartite interaction between Y139, Y342, and H375 at the SH2-kinase interface drives SH2-mediated resistance. While Y139C was previously identified with the tool molecule GNF-2 [118], this is the first evidence that the clinically approved small molecule asciminib also requires Y139 and adds new structure-function resolution by identifying an interaction between Y139, Y342, and H375. Together, our data are consistent with prior literature, but we also discover 279 novel resistance mutations and contribute to an unprecedentedly clear insight into the structure-function relationships governing asciminib activity in the myristoyl pocket, SH3, and SH2 domains. By combining base editing and deep mutational scanning our work expands upon prior findings by providing a more comprehensive understanding of the diverse resistance landscape that may emerge in the future and the underlying structural features that are responsible for this landscape.

We report, to our knowledge, the first documented example of an epistatic drug resistance screen using an “edit-vs-sgRNA library of edits” approach. Base editing allows for the introduction of clinically relevant mutations at endogenous sites in the genome in an efficient manner. After generating this background mutation it is easy to stably introduce a library of sgRNAs. Using this approach to discover an interaction between the SH3 domain and the P-loop underscores the power of an “edit-vs-sgRNA library of edits” approach to uncover novel molecular interactions. While the precise mechanism of the interaction between V73A and Y253H remains to be fully elucidated, our FRET data suggests that these mutations combine to dramatically open the ABL1 conformation. This observation aligns with previous reports demonstrating increased activity and a more open conformation for ABL1 Y253H using enzymatic assays [119] and nuclear magnetic resonance spectroscopy [103]. Additionally, introducing SH3 and SH2 domains to purified kinase domain have been shown to favor an “I_2_ inactive state” of ABL1 in NMR studies and this conformation is further stabilized by an asciminib analog [103]. Combining these key structural observations with our FRET and genetic data leads us to propose that the Y253H and V73A mutations destabilize the established equilibrium observed in foundational NMR studies, perhaps leading to a shift away from an “I_2_ like” inactive state that is less sensitive to asciminib binding. The identification of epistasis between the SH3 domain and the P-loop suggests that the emergence of two weakly resistant mutations can confer significant resistance to asciminib. However, we are also impressed by the rarity of this type of epistatic interaction in our screen. The V73A mutation was the only strongly epistatic mutation we identified. This finding may reflect a general principle of molecular evolution, as extensive studies of HSP90 [96, 120] and β-lactamase [30] also suggest that positive epistasis is uncommon.

One of our deepest findings is the strong correlation between ABL1 “openness” measured by FRET in live cells and the degree of cellular sensitivity to asciminib across 9 different asciminib resistance mutations in ABL1 (*r* = -0.82, Fig. 4D). Our FRET biosensor was designed with NMR studies [102, 103] in mind. The positioning of our fluorophores was informed by structural data that depicted an equilibrium between open and closed states of the protein [68, 68, 102, 103, 110]. However, these structural studies utilize purified proteins in solution, and our FRET assay is performed in live cells. We report the strongest evidence to date that an “open” conformation of ABL1, as measured with FRET, is present in live cells and that this state is quantitatively correlated with asciminib drug response in live cells. The high correlation coefficient suggests that this mechanism biophysically unifies single mutations in the myristate pocket, SH2 domain, SH3 domain and the P-loop as well as the epistatic combinations of a P-loop mutation and an SH3 domain mutation. All resistance mutations converge on this biophysical transition between closed and open conformations. This convergence in conformational mechanism across so many mutations at distinct positions in ABL1 is exciting because it implies that the solution to the asciminib resistance problem may only have to combat a single biophysical phenomenon.

While our flow cytometry-based FRET data strongly correlates with asciminib dose-response assays, FRET measurements are inherently influenced by factors such as fluorophore distance and orientation [113]. The observed differences in basal FRET signal, in the absence of asciminib suggests that the ABL1 mutations induce some baseline conformational changes, which may also alter the orientation of the fluorescent proteins, thereby affecting FRET efficiency in response to asciminib. Importantly, the observed trend remains consistent in a background-subtracted re-analysis of the FRET data (Additional file 1: Fig. S7). Despite these potential caveats, we confirmed the biological validity of our reporter: the FRET BCR::ABL1 construct successfully transforms Ba/F3 cells and, critically, does not significantly alter the asciminib resistance profile compared to cells expressing unmodified BCR::ABL1. Therefore, while the FRET data provides valuable insights, caution is warranted in drawing a direct line between the exact structures observed in biophysical studies and the FRET signal in our study.

## Conclusions

The emergence of drug resistance mutations in a drug target is a hallmark of on-target small molecule activity in patients. However, the expanding repertoire of active compounds with distinct resistance profiles allows for the design of sequential or combination therapies that can mitigate the likelihood of resistance emergence by using one molecule to plug the mutational gaps of a second molecule [121]. Yet this strategy is often employed ad hoc in clinical settings and dramatically expands the possibilities for resistance by varying drug composition, dose, order, and more. Toward that complexity, leveraging high-throughput functional genomics approaches, such as base editing and deep mutational scanning, across diverse genetic backgrounds offers a powerful approach to identify optimal therapeutic combinations and sequences by deconvoluting path-dependent resistance.

## Supplementary Information

Additional File 1. Supplementary Figures 1 – 7. Accompanying analysis and information in support of main figures.

Additional File 2. Base Editor Screens. Base editor screen sgRNA sequences, counts, and statistics.

Additional File 3. Deep Mutational Scanning Screens. Deep mutational scanning screen counts and statistics.

## Data Availability

To promote reproducibility, all raw data are available on figshare https://figshare.com/projects/Dual_functional_genomics_reveals_a_broad_and_convergent_landscape_of_asciminib_resistance_in_BCR_ABL1/274789 [75, 79, 84, 101, 104]. The custom code used for this analysis is publicly accessible on GitHub https://github.com/TheBioinformatician/Dual-Functional-Genomics-Asciminib [122, 123]. The raw sequencing data for this study have been deposited in the NCBI Sequence Read Archive under BioProject accession number PRJNA1307639 https://www.ncbi.nlm.nih.gov/bioproject/PRJNA1307639 [124]. Plasmids used in this study are available on Addgene https://www.addgene.org/browse/article/28253045/.

## Abbreviations

ABE: Adenosine base editing
ABL1: Abelson tyrosine-protein kinase 1
BCR: Breakpoint cluster region
CC: Coiled coil
DH: Dbl-homology
DMS: Deep mutational scanning
FAB: F-actin binding domain
FRET: Förster resonance energy transfer
LFC: Log2 fold change
MOI: Multiplicity of infection
PCR: Polymerase chain reaction
SH2: Src-homology 2
SH3: Src-homology 3
WT: Wild type
