# Supplementary material for: Dual functional genomics reveals a broad and convergent landscape of asciminib resistance in BCR::ABL1
Source: Genome Med. 2026 Jun 27;18:114. doi: 10.1186/s13073-026-01679-x (PMC13422289; doi:10.1186/s13073-026-01679-x)
Supplement: Supplementary file 1 — Additional File 1. Supplementary Figures 1 – 7. Accompanying analysis and information in support of main figures. [file 13073_2026_1679_MOESM1_ESM.docx]

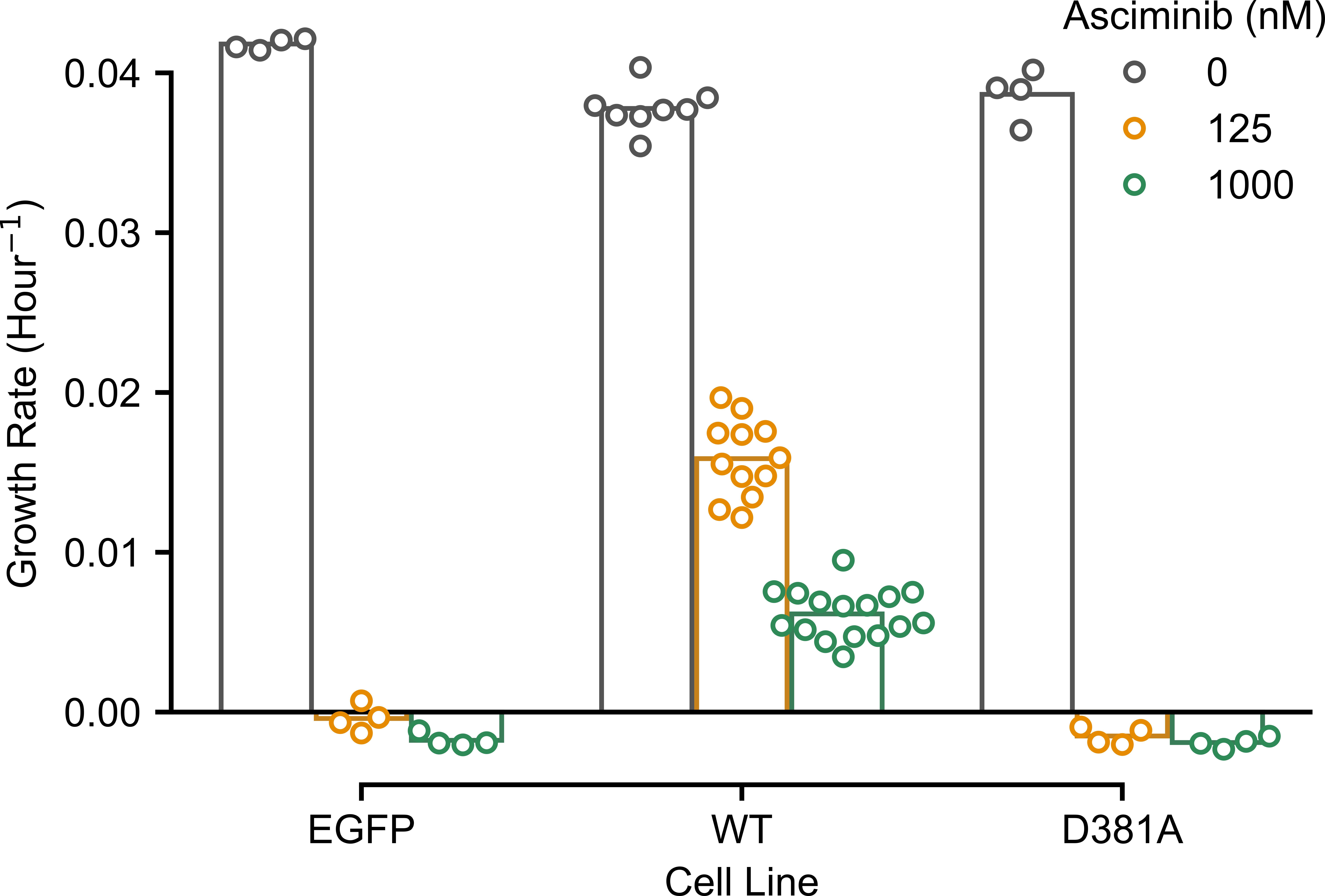


**Fig. S1** Growth rates of K562 cells overexpressing EGFP (control), WT BCR::ABL1, and BCR::ABL1 D381A (kinase-dead mutant) treated with asciminb or DMSO. (*N* ≥ 4)





**Fig. S2** High-quality adenosine base editing screens identify BCR::ABL1 resistance hotspots.

**A, B** Reproducibility of adenosine base editor screens. Scatter plots show the Pearson correlation coefficient (*r*) between biological replicates for **A** asciminib and **B** imatinib selection in K562 cells. Log2 fold change (LFC) values are plotted for individual guides. Guides targeting the genomic BCR::ABL1 locus are indicated in blue; control guides (including iSTOP, intergenic targeting, and non-targeting) are indicated in orange.

**C, D** Distribution of resistance-conferring mutations across the BCR::ABL1 protein. Scatter plots represent the mean LFC of guide abundance following treatment with **C** asciminib or **D** imatinib. Positive LFC values indicate enrichment and a gain in fitness/resistance. Structural domains of BCR::ABL1 are annotated as follows: coiled-coil (CC), Dbl-homology (DH), Pleckstrin-homology (PH), Src-homology 3 (SH3), Src-homology 2 (SH2), kinase, and F-actin binding domain (FAB).





**Fig. S3** Deep mutational scan quality control

**A** Pearson correlation (r = 0.92) between biological replicates of the deep mutational scan in K562 cells, demonstrating high reproducibility. (*N* = 2)

**B** Distribution of normalized growth rates of mutants detected by deep mutational scan in K562 cells. The dashed gray line represents -2 standard deviation (σ) from the sensitive distribution of variants in blue. Resistant variants are represented by the distribution in orange.

**C** Dot blot against ABL1 and Actin. All Ba/F3 BCR::ABL1 variants maintain WT-like expression levels. (*N* = 3)

**D** Distances between residues at the ABL1 SH2 kinase interface. (AlphaFold3 ABL1)

***Dot Blot Analysis of ABL1 and Actin***

Approximately 2 × 10^6^ Ba/F3 cells were washed with phosphate-buffered saline (PBS) and resuspended in 50 µL of PBS. The cells were lysed on ice for 3 minutes by adding 25 µL of 2% Triton X-100 in PBS followed by brief vortexing. Two microliters of the resulting lysate were spotted onto a nitrocellulose membrane and allowed to air dry. The membrane was blocked using 5% bovine serum albumin (BSA) in PBS.

For protein detection, the blot was incubated in a primary antibody solution containing 1:200 mouse anti-ABL1 (Santa Cruz, sc-56887) and 1:1000 rabbit anti-pan-actin (Cell Signaling Technology, #4968) in PBST (PBS with 0.1% Tween-20) supplemented with 2.5% BSA. Following three washes with PBST, the membrane was incubated for 2 hours with LI-COR IRDye anti-rabbit and anti-mouse secondary antibodies (1:1000). After three additional PBST washes, the blot was scanned using a LI-COR Odyssey Imager for the simultaneous detection of ABL1 and actin.





**Fig. S4** Generation of Imatinib-Resistant K562 Cells via Base Editing
**A** , **B** Schematic representation of base editing strategies for introducing **A** the ABL1 Y253H mutation using adenosine base editing, **B** the ABL1 T315I mutation using cytosine base editing, and **C** the ABL1 V73A mutation by adenosine base editing. Protospacer adjacent motif of each guide is color coded in forest green.

**D**, **E**, **F** Sanger sequencing confirmation of base-edited **D** K562 ABL1 Y253H cells, **E** ABL1 T315I cells, and **F** ABL1 V73A/Y253H cells. Note that the T315I guide also resulted in bystander editing of a synonymous I314I mutation. Similarly, V73A sgRNA creates a synonymous F72F mutation.

**G** Volcano plot showing differential guide frequency in K562 WT and Y253H cells following asciminib treatment. Positive log2 fold change (LFC) indicates enrichment of guides in K562 Y253H cells. Dashed oval highlights the two most significantly enriched guides. (*N* = 2)

**Table S1**. Dose response parameters of base edited K562s demonstrate epistasis. (*N* = 3*)*

| **K562 + Guide** | **Asciminib** | | **Imatinib** | | **Ponatinib** | |
| --- | --- | --- | --- | --- | --- | --- |
|  | *EC_50_* (nM) | *Bot* (%) | *EC_50_* (nM) | *Bot* (%) | *EC_50_* (pM) | *Bot* (%) |
| WT + AAVS1 | 4.2±0.3 | 0±1 | 95±4 | 0±1 | 180±10 | 1±2 |
| WT + V73A | 10.0±0.8 | 0±2 | 109±11 | 0±3 | 160±9 | 2±2 |
| Y253H + AAVS1 | 13.9±0.9 | 2±2 | >2000 | NA | 580±40 | 2±2 |
| Y253H + V73A | 32±3 | **25±2** | >2000 | NA | 650±30 | 2±1 |
| Y253H + V73A* |  | **3±7** |  |  |  |  |

*Theoretical *Bot* value based on Product model of gene interaction.

**Table S2**. Dose response *EC_50_* values of asciminib resistance mutations in Ba/F3 BCR::ABL1 cells. (*N* = 3*)*

| **Ba/F3** | ***EC_50_* (nM)** |
| --- | --- |
| + IL-3 | >500 |
| WT | 2.3±0.4 |
| V73A | 6.1±0.7 |
| Y253H | 5.0±0.8 |
| V73A Y253H | **40±30** |
| V73A Y253H* | **13±3** |
| M244V | 35±6 |
| V468F | >500 |

*Theoretical *EC_50_* based on Product model of gene variant interaction.





**Fig. S5** Characterization of the BCR::ABL1 FRET sensor.

**A** Schematic representation of the BCR::ABL1 FRET sensor cDNA construct.

**B** Excitation and emission spectra of mStayGold and mScarlet3 (adapted from FPbase). The shaded areas indicate the filter sets used in the flow cytometer to detect the respective fluorophore emissions.

**C** Validation of FRET Pair and Asciminib-Induced Conformational Response. Mean FRET ratios measured in HEK293T cells following treatment with 1 uM asciminib (orange) or vehicle control (DMSO, gray). To validate the specificity and intramolecular nature of the reporter, several controls were utilized: the ABL1 FRET construct (double-fluorophore) confirms the intramolecular response, while single-fluorophore constructs (ABL1 mStayGold, ABL1 mScarlet3) account for spectral background, and the absence of intermolecular FRET response. The Short Fusion construct serves as a high-FRET structural control. The full ABL1 FRET biosensor demonstrates a robust increase in FRET ratio upon asciminib treatment, indicating a successful conformational shift toward the closed, inactive state. (*N* ≥ 3)

**D** Asciminib dose response curves for HEK293T cells expressing the ABL1 FRET biosensor with wild-type (WT) ABL1 or the indicated asciminib resistance mutations in SH3 domain or SH2-kinase interface. (*N* = 3)





**Fig. S6** Independent FRET positive control

**A** Schematic representation of the SHP2 FRET sensor cDNA construct.

**B** Schematic representation of batoprotafib's effect on SHP2 E76G conformational dynamics. The activating mutation E76G shifts SHP2 to an open, active conformation. Batoprotafib treatment promotes a return to the closed, inactive conformation, thereby reversing the oncogenic activation. (SHP2 AlphaFold3)

**C** Mean FRET ratios from HEK293T cells expressing the SHP2 FRET biosensor. Cells expressing the activating E76G mutation show a lower FRET ratio compared to wild-type (WT) SHP2, indicative of an open conformation. Batoprotafib (1 uM) treatment increases the FRET ratio in E76G mutants, suggesting a shift towards a more closed, inactive state. (*N* ≥ 2)





**Fig. S7** Re-analysis of FRET biosensor data accounting for basal signal differences.

**A, B** Re-analysis of asciminib dose-response curves for ABL1 FRET biosensor activity in HEK293T cells (corresponding to data in Fig. 5C and Fig. S5D, respectively). Data are plotted as Adjusted (Adj.) Mean FRET, calculated by subtracting the basal FRET ratio (DMSO) from the treated values to normalize for baseline conformational variations. (*N* = 3)

**C** Scatter plot showing the Pearson correlation between asciminib *EC_50_* values in Ba/F3 cells expressing BCR::ABL1 and the adjusted mean FRET ratios in HEK293T cells treated with 100 nM asciminib. (*N* = 3)
